# Maternal Lifestyle During Pregnancy and Its Influence on Offspring’s Telomere Length

**DOI:** 10.3390/life15081250

**Published:** 2025-08-06

**Authors:** Elena Vakonaki, Maria Theodora Vitiadou, Eleftherios Panteris, Manolis Tzatzarakis, Aristides Tsatsakis, Eleftheria Hatzidaki

**Affiliations:** 1 Laboratory of Toxicology, School of Medicine, University of Crete, 70013 Heraklion, Greece; evakonaki@med.uoc.gr (E.V.); mvitiadou9@gmail.com (M.T.V.); tsatsaka@uoc.gr (A.T.); 2 Department of Neonatology and NICU, University General Hospital of Heraklion, School of Medicine, University of Crete, 70013 Crete, Greece; eleftherios.panteris@gmail.com

**Keywords:** telomeres, maternal lifestyle, maternal nutrition, physical activity, fetal programming

## Abstract

Telomeres are protective DNA sequences located at chromosome ends, essential to maintaining genomic stability. This narrative review examines how maternal lifestyle factors during pregnancy influence fetal telomere length (TL). Positive associations have been identified between offspring’s TL and maternal consumption of nutrients such as vitamins C and D, folate, and magnesium. Additionally, adherence to a Mediterranean diet and regular physical activity during pregnancy are correlated with increased placental TL, supporting fetal genomic integrity. Conversely, maternal dietary patterns high in carbohydrates, fats, or alcohol, as well as exposure to triclosan and sleep-disordered breathing, negatively correlate with offspring’s TL. Maternal infections may also shorten TL through heightened inflammation and oxidative stress. However, evidence regarding the impact of other lifestyle factors—including maternal stress, smoking, caffeine intake, polyunsaturated fatty acid consumption, obesity, and sleep quality—remains inconsistent. Given that shorter telomere length has been associated with cardiovascular, pulmonary, and neurodegenerative diseases, as well as certain types of cancer, these findings highlight the vital importance of maternal health during pregnancy in order to prevent potential adverse effects on the fetus. Further studies are required to elucidate the precise timing, intensity, and interplay of these influences, enabling targeted prenatal interventions to enhance offspring health outcomes.

## 1. Introduction

Telomeres are noncoding double-stranded tandem repeats (5′-TTAGGG-3′) located at the ends of chromosomes that play a key role in preserving genomic stability and integrity [1]. They safeguard chromosomes from excessive DNA loss during replication and help buffer against oxidative stress-induced damage [2]. Telomere length (TL) is at its greatest during early fetal development and already begins to shorten before birth [1,3]. With each cell division, TL undergoes gradual attrition, reaching a critically short length—known as the Hayflick limit—which triggers cell-cycle arrest, which culminates in apoptosis or senescence, observed primarily in in vitro culture, depending on the cell type and stress environment [4].

Several methods have been developed to quantify TL, with quantitative polymerase chain reaction (Q-PCR) assays widely adopted for their efficiency and accuracy [5]. Statistical models based on Q-PCR data have enhanced the interpretation of TL measurements, often using peripheral blood leukocytes from adult populations [6,7]. In population-based studies, TL is typically categorized by percentile distribution: lengths below the 10th percentile are considered short and those above the 90th percentile long [6].

**Figure 1:**
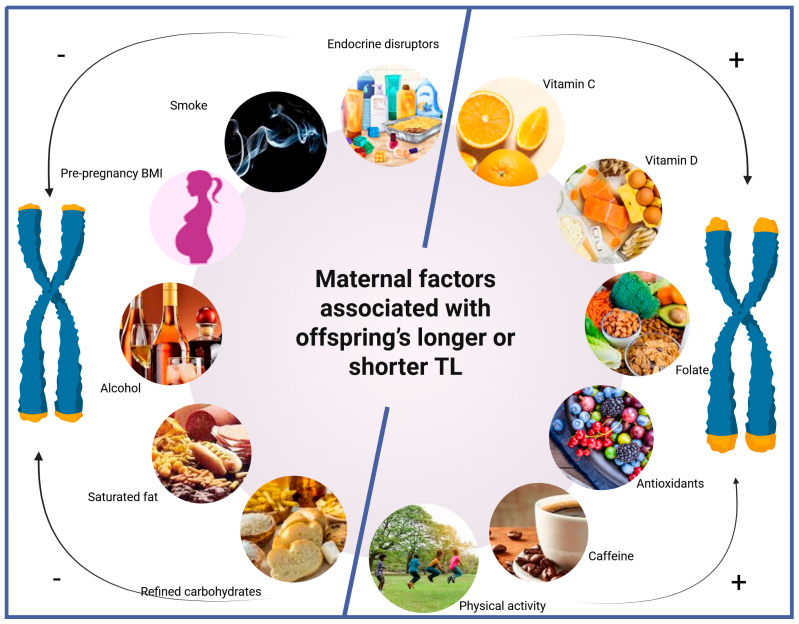
Maternal lifestyle and environmental factors shape telomere length at birth (Created in BioRender. Tsatsakis, A. (2025) https://BioRender.com/big47rd, accessed on 4 August 2025).

Peripheral blood leukocyte TL is a recognized biomarker of ageing and age-related disease, whereas placental and other mitotically active tissues show correlated lengths, while postmitotic (PM) tissues have a much stronger inheritance component and do not shorten during adult life [8]. In neonates, however, cord-blood and placental samples provide baseline measures of fetal telomere biology [9]. These tissues are non-invasive to collect and offer insights into fetal telomere dynamics. While the umbilical cord is purely fetal in origin, placental tissue comprises both fetal and maternal components [10]. Notably, placental TL appears more susceptible to certain maternal influences—such as nutritional status—compared with cord-blood TL [11]. Emerging studies suggest that this biological vulnerability window in utero may have lasting implications for the offspring’s future health, depending on the quality of the intra-uterine environment. The concept that prenatal exposures shape long-term disease risk aligns with the “Developmental Origins of Health and Disease” (DOHaD) framework, which suggests that fetal development is highly plastic and sensitive to maternal cues such as diet, stress, and environmental toxins. Telomere biology is now increasingly recognized as one of the cellular mechanisms mediating this developmental programming [12]. Figure 1 shows the most common maternal lifestyle and environmental factors that can affect telomere length.

This review aims to explore how key maternal lifestyle factors during pregnancy—including nutrition, physical activity, stress, obesity, sleep, smoking, and environmental exposures—may influence TL in the offspring and shape long-term health trajectories.

Given the narrative nature of this review, a formal systematic search protocol was not applied. Instead, relevant studies were identified through manual searches on PubMed and Google Scholar using combinations of terms such as telomere length, maternal lifestyle, pregnancy, nutrition, fetal programing, and oxidative stress. Preference was given to peer-reviewed articles published in the past 10 to 15 years that offered meaningful insights into the relationship between prenatal factors and telomere dynamics. While not exhaustive, the selection reflects the most current and pertinent findings available at the time of writing.

## 2. Biological Role and Regulation of Telomeres in Fetal Life

### 2.1. Telomere Dynamics in Fetal Development

Telomere length varies significantly among individuals of the same age, and this variability is already evident at birth. Initial TL is particularly important, as it influences subsequent telomere attrition rates; paradoxically, a longer TL at birth often associates with faster attrition during childhood [13]. Interest in fetal and neonatal telomere biology has grown, largely due to its implications for long-term health. During key periods of cellular proliferation and differentiation, interactions between the intra-uterine environment and genetic programming can modulate TL regulation [4,14]. These early modifications can influence disease susceptibility throughout life, either on their own or in interaction with later-life environmental exposures [15]. However, these associations are largely drawn from observational research, and causal relationships remain difficult to establish due to confounding variables and heterogeneity in TL measurement techniques.

TL at birth is strongly predictive of TL across the lifespan [16], and inter-individual differences remain relatively stable with age. Notably, women tend to have longer telomeres than men, a pattern observed in several birth cohorts [16,17]. Shorter TL in fetuses has been associated with increased risk for cardiovascular conditions [18,19], as well as later-life metabolic and pulmonary disorders, including diabetes [20]. In contrast, longer TL has been linked to more favorable outcomes, such as lower childhood blood pressure [16], although it may also be associated with greater susceptibility to cancer tumorigenesis [21]. Both short and long telomeres have been associated with cancer risk, depending on the type of cancer, possibly reflecting different mechanisms of telomere dysfunction [22,23]. This paradox arises because, while shorter telomeres are often markers of cellular ageing and reduced regenerative capacity and are linked with genomic instability, longer telomeres may permit unchecked proliferation, thereby facilitating tumorigenesis [21,22]. Therefore, TL may act as a double-edged sword—its interpretation depends on clinical context and life stage.

Thus, while TL has clear clinical associations, it should be interpreted as a biological vulnerability marker rather than a direct causal agent in disease development. TL is increasingly viewed as both a potential biomarker for disease susceptibility and a target for therapeutic exploration in certain pediatric and chronic conditions [2]. Ultimately, an individual’s TL later in life is shaped by their initial TL at birth and the early-life rate of attrition [4]. These concepts align with Barker’s hypothesis [24] and the broader DOHaD framework [12], which suggests that environmental conditions during critical prenatal and perinatal periods program long-term disease risk, including cardiovascular disease, type 2 diabetes, stroke, and obesity [25]. Barker’s hypothesis suggests that sub-optimal fetal environments permanently alter organ structure and physiology, predisposing the individual to chronic disease in adulthood [24]. While promising, this area of research requires harmonized protocols for telomere assessment across tissues and cohorts to enhance reproducibility and generalization of findings.

### 2.2. Genetic and Maternal Inheritance of TL

TL regulation begins before birth, with evidence showing a strong correlation between maternal and neonatal TL. Genome-wide association studies have identified multiple genetic variants implicated in telomere inheritance across generations [13]. These heritability patterns appear more pronounced in females and are also observed among siblings [26]. Specific chromosomal loci further support the polygenic nature of TL regulation [27,28]. Nevertheless, genetic effects do not act in isolation and may be moderated by intra-uterine and early postnatal environments.

While paternal age has often been cited as a key factor in TL inheritance, sperm telomere length tends to increase with advancing paternal age, likely because telomerase remains active in male germ cells or due to a recombination-based alternative lengthening of telomeres, thereby conferring longer telomeres to offspring conceived later in a father’s life [7,29,30]. Emerging studies also point to the importance of maternal contributions—possibly via X-linked genetic mechanisms [31]. Heritability estimates for TL range from 36% to 82%, even in neonatal populations, indicating a strong genetic component modulated by environmental influences [30].

As the intra-uterine environment is largely shaped by maternal physiology and behavior, pregnant women play a critical role in determining fetal TL and, consequently, the future health trajectory of their offspring [12,21,24]. Maternal age, for instance, has been identified as a determinant of TL, with recent findings suggesting that children born to mothers over age 29 may have longer telomeres compared with those born to younger mothers—although the molecular underpinnings of this observation remain unclear [32,33].

Beyond age, maternal lifestyle factors such as physical activity, sleep quality, smoking, and stress exposure all appear to influence fetal TL, with healthier behaviors associated with longer telomeres [18,34,35]. Nutritional status is also critical: a balanced maternal diet supports optimal fetal growth and can either lengthen or shorten TL depending on specific nutrient profiles [25,36,37]. Conversely, exposure to environmental pollutants and chemical toxins during pregnancy has been shown to impair TL maintenance in the fetus, potentially increasing susceptibility to chronic disease [38]. The large-scale flow-FISH profiling of an adult population cohort has recently shown that beyond inherited genetics, parental age (both maternal and paternal), smoking, and other lifestyle exposures independently explain significant inter-individual variation in leukocyte telomere length, highlighting the importance of modifiable behaviors in telomere biology [39].

## 3. Maternal Lifestyle Factors Influencing Telomere Length

### 3.1. Nutrition and Dietary Patterns

Maternal nutrition influences fetal telomere length (TL) through intertwined effects on DNA synthesis, oxidative balance, inflammation, and epigenetic programming [36,40]. Accordingly, both the quality of individual nutrients and the overall dietary pattern adopted in pregnancy can accelerate or attenuate telomere attrition in utero. However, most findings stem from observational studies, and causality cannot be assumed without accounting for residual confounding, especially socioeconomic and lifestyle factors correlated with diet quality.

A principal mechanistic link is oxidative stress. Telomeres, rich in guanine, are especially vulnerable to reactive oxygen species (ROS). Vitamin C, a potent water-soluble antioxidant that is often supplemented during pregnancy, scavenges free radicals and stabilizes telomeric DNA; higher maternal concentrations correlate with longer neonatal TL, whereas deficiency is associated with chromosome fragility, impaired telomerase function, accelerated telomere shortening and a greater risk of complications such as pre-eclampsia [36,41,42,43,44]. Despite supportive correlations, vitamin C intervention trials in pregnant populations assessing telomere-related endpoints remain lacking.

Vitamin D provides complementary protection. Because the fetus cannot synthesize it, 25-hydroxyvitamin D must cross the placenta in adequate amounts [45]. Low maternal status heightens systemic inflammation and oxidative stress, both of which favor telomere shortening in cord-blood cells [36,46]. Folate supports nucleotide synthesis and DNA methylation; each 10 ng dL^−1^ rise in early-pregnancy folate has been linked to a 5.8% increase in newborn TL [47]. Beyond these vitamins, adequate magnesium intake has been associated with longer TL in cell-free amniotic DNA, whereas vitamin B_1_ and iron show weaker, non-significant trends in similar directions [48]. Animal studies further demonstrate that sufficient methyl-donor availability maintains telomere epigenetic stability [49].

The macronutrient profile also matters. High-glycemic carbohydrates—such as white bread, refined cereals, and sugar-sweetened beverages—consistently predict shorter TL in cord blood [50,51,52], although total carbohydrates providing roughly 47–70% of energy supports normal fetal growth [53]. Diets rich in saturated fat—from full-fat dairy or processed foods—are likewise associated with telomere attrition [36,54,55]. Low maternal n-3 polyunsaturated fatty acid (PUFA) status and a high n-6:n-3 ratio shorten cord-blood TL, yet very high placental n-3 levels have paradoxically coincided with telomere loss, highlighting tissue-specific thresholds [2]. Transient caloric restriction in animal models improves mitochondrial efficiency, reduces ROS, and preserves telomeres, although human evidence remains confined to adult studies [12]. These findings illustrate the importance of dosage, timing, and tissue specificity, which remain underexplored in pregnancy cohorts.

Protein associations are mixed: Higher third-trimester intake lowers pre-term-birth risk and lengthens gestation [56], but it has also been linked to lower birth length and slower early growth [57]. Observational studies disagree on red-meat protein, where some implicate oxidative stress and shorter TL [58,59], whereas others report neutral or favorable outcomes when the overall diet is balanced [60].

Because nutrients act synergistically, whole-diet models provide clearer insights than single-nutrient analyses. The Mediterranean diet (MD)—characterized by abundant fruits, vegetables, legumes, whole grains, nuts, and extra-virgin olive oil, with modest fish and limited processed meat—improves pregnancy outcomes, including lower risks of gestational diabetes, urinary-tract infection and pre-term birth [61,62]. Adult cohorts adhering to the MD exhibit longer TL and greater telomerase activity [63]. In children and adolescents, MD-aligned food choices—fish, legumes, nuts, and unsaturated fats—correlate with longer TL, whereas processed foods, refined grains, and added sugars predict shortening [51]. Regarding later years, particularly in children and adolescents, research indicates that consuming more fish, nuts, seeds, fruits, vegetables, olives, legumes, and PUFAs and having higher total antioxidant capacity (TAC) correlate with longer telomeres [51]. However, null or inverse associations have also been reported, likely due to cultural variability in what constitutes a “Mediterranean” diet and potential residual confounding [64,65].

Plant-based diets (vegan and lacto-ovo vegetarian) provide a second composite model. When centered on whole grains, legumes, nuts, seeds, vegetables, and fruits, they supply high fiber, antioxidants, phytochemicals, and folate, which can enhance DNA repair and modulate methylation [66,67,68]. Some reports link such patterns to longer TL [69,70]. Others observe no difference or—even when refined plant foods dominate—shorter TL and heightened inflammation, effects most pronounced in non-Hispanic white cohorts [71,72,73]. Such discrepancies may arise from differences in plant-based diet quality, nutrient bioavailability, or the underreporting of supplement use. Nutritional adequacy remains paramount: iron, zinc, vitamin B_12_, iodine, calcium, vitamin D, and long-chain omega-3 fatty acids may be marginal in strict vegan regimens [74]. The American Dietetic Association considers well-planned vegetarian diets appropriate throughout pregnancy, whereas the German Nutrition Society urges caution with exclusive veganism owing to potential micronutrient deficits [67,75]. Pregnant women are, therefore, advised to consume a varied food repertoire and increase total energy intake during the second and third trimesters to meet rising nutrient demands [66].

Collectively, current evidence suggests that diets rich in minimally processed, antioxidant-dense foods balanced in unsaturated fats are the most conducive to preserving fetal TL, whereas patterns dominated by refined carbohydrates, saturated fats, or uncorrected micronutrient deficiencies appear to accelerate telomere erosion. However, given the predominance of observational data and lack of harmonized nutritional metrics across studies, these findings should be interpreted with caution.

### 3.2. Physical Activity

Research examining physical activity (PA) and telomere length (TL) in pregnancy remains scarce. Most investigations have analyzed maternal rather than neonatal telomeres, and only a handful have addressed offspring outcomes directly [76]. Studies that do involve neonates have largely measured overall well-being rather than TL itself [76,77,78]. To date, a single randomized trial has measured placental TL immediately after delivery and found no difference between exercise and control groups despite the well-documented antioxidant and anti-inflammatory benefits of maternal exercise for both mother and child [34]. These null findings may be attributable to small sample sizes, variability in TL assay methods, or a lack of adjustment for diet and other lifestyle confounders.

Proposed mechanisms by which PA could protect telomeres include up-regulation of endogenous antioxidant enzymes [79], enhancement of DNA-repair pathways [80], stabilization of shelterin proteins that cap chromosome ends [81], and reduction in reactive oxygen species production [82]. Although definitive human data during gestation are lacking, exercise consistently shows favorable TL trends—particularly when combined with a nutrient-dense diet [37,83]. The GESTAFIT project reported longer placental telomeres among women who exercised regularly while adhering to a Mediterranean dietary pattern, suggesting potential synergy between dietary quality and PA [34]. This remains an isolated finding and has yet to be replicated in larger, multi-center trials.

Clinical guidelines emphasize that pregnancy is not a time for complete rest. The American College of Obstetricians and Gynecologists and the U.S. Department of Health and Human Services recommend at least 150 min of moderate-intensity aerobic exercise per week, distributed over several days [84] (Table 1). Women who were highly active before conception may continue most activities, though anatomical and physiological changes often necessitate adjustments in frequency, duration, or intensity. Consensus is still lacking on how much strenuous exercise is optimal, and its precise impact on perinatal outcomes remains uncertain [18]. Inter-individual variability in pregnancy adaptation may influence both adherence and biological response to exercise, complicating efforts to standardize recommendations for specialists.

Within recommended limits, greater frequency, duration, or volume of PA generally confers larger benefits, yet current evidence does not show additional safety or advantage when activity far exceeds guidelines. Safe options during pregnancy include brisk walking, stationary cycling, low-impact aerobics, dance, stretching, and light resistance training with weights or elastic bands [88]. Early-gestation, low-intensity exercise may enhance placental perfusion [76] and appears to reduce risks of pre-eclampsia and pre-term birth, although many findings do not reach statistical significance [89]. Small sample sizes, differing endpoints, and inadequate reporting on exercise modality limit the generalizability of many such results.

Large-cohort analyses and national recommendations indicate that prenatal PA is not associated with miscarriage, stillbirth, congenital anomalies, or other adverse events. A meta-analysis of more than 200 000 participants showed that 600 Metabolic Equivalent of Task (MET)-minutes per week of moderate activity lowers the risk of gestational diabetes by roughly 25% [90]. As summarized in recent Canadian and European position statements, PA is advised to reduce pregnancy complications and support maternal physical and mental health [91].

Despite these encouraging data, evidence linking maternal exercise directly to offspring TL is still insufficient. Many trials report positive trends without statistical significance, often because of limited sample size, wide variability in TL assessment methods, inadequate dietary control or the inclusion of only healthy women. Future studies should incorporate supervised exercise protocols, detailed nutritional monitoring, and more diverse populations to clarify the influence of prenatal PA on neonatal TL and long-term cardiometabolic outcomes [76].

## 4. Maternal Stressors and Systemic Inflammation

Several maternal conditions—including obesity, psychological stress, and infection—share convergent biological pathways that may influence fetal telomere length. Central to all three is a state of chronic low-grade inflammation and heightened oxidative stress, which can accelerate telomere attrition in placental and fetal tissues [4,92]. These exposures often co-occur and may act synergistically, amplifying cellular ageing processes through excess production of reactive oxygen species, immune activation, and disruption of hormonal signaling [15,36]. Moreover, obesity and stress are associated with metabolic dysregulation and impaired placental function [40,93], while infections may compromise immune tolerance and provoke fetal inflammatory responses [92,94]. Given these overlaps, it is biologically plausible that these conditions exert cumulative effects on fetal telomere biology, warranting their joint consideration in both clinical risk assessment and mechanistic studies [16,19].

### 4.1. Obesity

Being overweight or obese during pregnancy is widely acknowledged to increase the risk of obstetric complications [95]. Maternal overweight is defined as a body mass index (BMI) of 25.0–29.9 kg m^−2^, whereas maternal obesity is a BMI ≥ 30 kg m^−2^ and is further categorized into class I (30–34.9), class II (35–39.9), and class III (≥40) [78]. Higher maternal BMI is accompanied by elevated blood glucose levels and a greater likelihood of pre-eclampsia, gestational diabetes, and hypertensive disorders [96]. First-trimester BMI is also correlated with an elevated risk of childhood obesity at age 4, likely reflecting in utero metabolic programming [95,97].

With respect to telomere biology, several studies suggest that higher maternal BMI—especially in the overweight or obese range—may be associated with shorter fetal telomeres [98]. Martens et al. documented that every one-unit increase in maternal BMI corresponded to ~50 bp shorter telomeres in umbilical-cord-blood cells [96], while Maugeri et al. found an inverse relation between gestational weight gain and the telomere length of cell-free DNA in early pregnancy [99]. However, sample sizes are often modest, and effect sizes vary depending on tissue type, telomere measurement method, and the timing of BMI assessment.

Although the precise mechanism remains unclear, chronic low-grade inflammation and heightened oxidative stress during fetal development are the leading candidates [98]. Pre-pregnancy BMI has likewise been associated with shorter placental and cord-blood telomeres [96]. Sex-stratified analyses reveal that the effect of higher maternal BMI is more pronounced in male newborns, potentially because estrogen in female fetuses up-regulates antioxidant enzymes, thereby mitigating oxidative insult [100,101]. Recognizing these risks, prenatal-care guidelines emphasize appropriate gestational weight gain: 7–11 kg for women who enter pregnancy overweight (BMI 25–29.9) and 5–9 kg for those with obesity (BMI ≥ 30) [102]. By maintaining weight gain within these ranges and attenuating the associated pro-inflammatory milieu, clinicians may help preserve offspring telomere integrity and reduce long-term metabolic risk. Nevertheless, no interventional studies to date have assessed whether improving maternal BMI or modifying gestational weight gain can causally influence neonatal TL.

### 4.2. Maternal Psychological Stress

Maternal psychological stress—manifesting as anxiety, depression, or elevated perceived-stress scores—has been linked to shorter telomeres in offspring [35,103]. Prenatal stressors that include significant life events or socio-environmental adversity have likewise been associated with fetal TL [104]. The timing, intensity, and chronicity of stress are important; long-term exposure appears to have the strongest effect on telomere shortening [103].

Stress regulation begins with the hypothalamic–pituitary–adrenal (HPA) axis [105]. When stress is perceived, the hypothalamus releases corticotropin-releasing hormone (CRH), prompting pituitary secretion of adrenocorticotropic hormone (ACTH); ACTH stimulates the adrenal glands to release cortisol, the primary stress hormone that helps the body adapt. Negative feedback ordinarily suppresses CRH and ACTH once cortisol reaches a threshold, but chronic stress can disrupt this loop, generating persistently high cortisol concentrations and adverse physiological effects [105]. Such dysregulation may increase oxidative stress and inflammation, thereby shortening telomeres. Elevated maternal cortisol crosses the placenta and can affect the fetus [106,107]. Recent findings indicate that individuals with heightened cortisol responses have immune cells with reduced capacity to up-regulate telomerase activity [4].

Maternal stress can negatively influence several fetal development pathways by inducing oxidative stress, inflammatory responses, and hormonal alterations during intra-uterine life [108]. Five cohort studies have reported shorter cord-blood TL in newborns whose mothers experienced higher perceived stress during pregnancy [93,103,107,109,110]. Entringer et al. first examined 27 mother–infant pairs and found that pregnancy-related stress evaluated early in gestation was linked to shorter neonatal TL [103]. Two larger studies (318 and 656 dyads) similarly observed that increased maternal perceived stress correlated with shorter telomeres in newborns [93,110]. In the latter, high maternal psychological resilience appeared to buffer this association, emphasizing the value of supporting maternal mental health [110]. In contrast, a cohort of 1405 newborns reported no association between maternal stress and TL [111], and Bosquet Enlow et al. observed that higher maternal stress was linked to longer telomeres among male newborns [112].

Sex-specific patterns, therefore, remain mixed. Some investigations show longer TL in girls at birth [93,113], whereas others report no significant sex difference [103,111]. Stressors such as financial strain or an unplanned pregnancy have been associated with shorter TL in females but longer TL in males [113]. These findings may be linked to post-birth attrition rates modulated by estrogen—which can boost telomerase activity and lower oxidative stress [101]. Although diverse stressors trigger well-characterized physiological responses, the molecular pathways by which they influence TL remain poorly delineated. Greater use of validated stress instruments, biological mediators (e.g., cortisol and cytokines), and repeated TL measurement over time will be critical to advancing this field.

### 4.3. Sleep

Sleep is deeply connected to stress, as disruption of sleep can exacerbate HPA axis dysregulation and inflammation and frequently co-occurs with psychological distress [114]. Sleep disturbance is common in pregnancy, particularly during the third trimester when physiological and hormonal changes intensify discomfort [115]. A recent meta-analysis estimates that between 30% and 75% of pregnant women experience poor sleep quality [114]. Sub-optimal sleep is associated with low birthweight, small-for-gestational-age infants, pre-eclampsia, and pre-term delivery. Sleep-disordered breathing (SDB)—most notably obstructive sleep apnea—further increases the risks of pre-eclampsia, neonatal-intensive-care admission, cesarean delivery, low birthweight, pre-term birth, small-for-gestational-age infants, and low Apgar score (a standardized clinical tool used to assess the condition of a newborn infant immediately after birth) [116].

Evidence linking maternal sleep to offspring telomere biology remains limited, as these studies offer preliminary support for a mechanistic link between maternal sleep and fetal TL but are based on small, demographically narrow cohorts. The first study to examine this question assessed sleep apnea risk and daytime sleepiness in pregnancy and found that newborns of mothers with high SDB risk had shorter telomeres, suggesting that sleep-related hypoxia and oxidative stress may accelerate in utero telomere attrition [116]. A second investigation reported that prolonged sleep duration coupled with poor subjective sleep quality in late gestation was likewise associated with shorter neonatal TL [115]. In contrast, a Finnish cohort of 1405 mother–infant pairs found no association between maternal insomnia and cord-blood TL [111]. Because current findings are inconsistent—and rely heavily on self-reported sleep metrics—more large studies using objective measurements (e.g., actigraphy or polysomnography) are needed to determine whether specific sleep characteristics, timing, or severity, influence fetal TL and to clarify the mechanisms involved.

### 4.4. Maternal Infection

Research on prenatal infection and offspring telomere biology is still sparse, yet emerging data suggest that maternal systemic inflammation can shape telomere length (TL) before birth [92]. During infection, heightened immune activity elevates pro-inflammatory cytokines such as tumor-necrosis factor-α (TNF-α) and interleukin-6 (IL-6) within the uterine environment [92]. Several pathways may convey these signals to the fetus: (i) transplacental passage of maternal cytokines and reactive oxygen species, (ii) local cytokine production within the placenta, and (iii) direct transfer of bacteria or lipopolysaccharide fragments across the placental barrier [94].

Both viral and bacterial pathogens are common in pregnancy and can influence fetal telomeres. Human herpesvirus-6 (HHV-6) provides a notable example: The virus can integrate into subtelomeric regions of parental chromosomes through homologous recombination, and about 1% of neonates carry chromosomally integrated HHV-6 [117]. Approximately 86% of chromosomally integrated HHV-6 cases reflect germ-line transmission from an infected parent; the remainder result from transplacental infection during gestation. Such integration has been linked to telomere dysfunction and reduced chromosomal stability in both mother and fetus.

Bacterial infection exerts comparable effects via oxidative and inflammatory stress. In a Southeast Asian cohort, group-B streptococcus infection correlated with shorter cord-blood TL; the association was the strongest when infection co-occurred with maternal anemia or hypertension, delivery complications, or neonatal jaundice—pointing to a synergistic burden of multiple stressors [94]. Experimental work corroborates these observations: pregnant mice injected with TNF-α displayed offspring telomere shortening across several tissues [118], and a follow-up study showed that TNF-α up-regulated activation transcription factor-7 (ATF7), a mediator of telomere erosion [119].

Collectively, these findings highlight the importance of early detection and treatment of maternal infections to limit inflammatory and oxidative insults that could accelerate telomere shortening in the developing fetus, potentially increasing long-term disease risk.

## 5. Smoking, Alcohol, and Caffeine

### 5.1. Smoking

Smoking during pregnancy transfers nicotine, carbon monoxide (CO), and more than 4000 additional toxic or carcinogenic compounds across the placenta [120]. Fetal telomere erosion appears to arise through several converging mechanisms: oxidative stress, inflammation, and altered mitochondrial function [121,122]. CO binds heme-containing proteins, including mitochondrial cytochrome-c oxidase, impairing cellular respiration and inducing tissue hypoxia [121]. Tobacco exposure also elevates free-radical production in both mother and fetus [122], and smoking has been clearly shown to have a dose-dependent relationship with female TL [123].

Empirical data remain limited but largely concordant. Five human studies report shorter telomeres in neonates exposed to maternal smoking in utero—both at birth [124,125,126,127,128] and into adolescence [127,128]. Only one investigation noted longer neonatal TL in the smoking group [129]. Second-hand smoke is likewise associated with shorter cord-blood telomeres, though values are still higher than in active-smoking dyads [124]. Although sample sizes vary and most studies are observational, the consistency of telomere shortening across cohorts strengthens the association.

According to Barker’s Developmental Origins hypothesis and the DOHaD framework, prenatal tobacco exposure may predispose the child to diseases linked to premature telomere attrition [130]. Indeed, fetal telomere loss has been connected to abnormal lung development and later pulmonary disorders such as idiopathic pulmonary fibrosis, emphysema, COPD, and lung cancer [8]. Maternal smoking has also been associated with a higher likelihood of ADHD in children [131]. Despite these risks, a recent U.S. survey showed that roughly one in fourteen women continued smoking during pregnancy and many more were exposed to passive smoke, highlighting an ongoing public health challenge [131].

### 5.2. Alcohol

Alcohol consumption is frequently included as a confounding variable in telomere research [132]. To date, most insights come from cell culture or animal models, with limited translation to human pregnancy. Large observational results are mixed: the largest study (>4500 participants) found no TL association, whereas smaller cohorts linked heavy drinking and alcohol use disorder to telomere shortening [133]. Clinical guidelines agree that there is no safe window for alcohol in pregnancy, urging abstinence from conception through delivery [134]. Alcohol can cause miscarriage, premature birth, and low birthweight—risks most pronounced in the first trimester [132]. Ethanol and its primary metabolite acetaldehyde (AcH) cross the placenta and accumulate in fetal blood at maternal levels; AcH, rather than ethanol itself, is chiefly responsible for telomere erosion in vitro [132,135]. Harpaz et al. outlined several molecular pathways through which AcH accelerates telomere loss, but further experimental work is needed to confirm these mechanisms in vivo [136]. Only one study has examined prenatal alcohol and offspring TL directly. That 2021 investigation showed that first-trimester drinking did not affect maternal leukocyte TL but did shorten telomeres in cord-blood DNA, reinforcing the DOHaD principle that early exposures can increase health risks later in life [132].

### 5.3. Caffeine

Caffeine, a component of coffee, tea, soft drinks, chocolate, and many over-the-counter remedies, crosses the placenta, and its clearance is 1.5–3.5 times slower in pregnancy [137,138,139]. From a cellular-signaling perspective, caffeine may activate PI3-kinase-related kinases (Tel1 and Mec1), proteins involved in telomere maintenance [137]. In a pilot study of 57 multiracial mother–newborn dyads, daily intake > 200 mg correlated with longer cord-blood telomeres [137]. This counterintuitive finding contrasts with the known prooxidant effects of high caffeine intake and should be interpreted cautiously due to the small sample and lack of replication.

Because epidemiologic evidence is sparse and derived from small samples, larger studies are required. Current public health recommendations prioritize overall pregnancy outcomes rather than telomeres: the World Health Organization advises < 300 mg caffeine day^−1^, while the ACOG recommends < 200 mg to minimize risks of miscarriage and low birthweight [140,141]. Until more robust data emerge, caffeine’s influence on fetal TL should remain a secondary consideration behind established obstetric safety thresholds.

## 6. Environmental Exposures

Emerging evidence shows that prenatal exposure to airborne pollutants and common consumer-product chemicals can affect fetal TL. The compounds most frequently examined—polycyclic aromatic hydrocarbons (PAHs), phthalates, and triclosan (TCS)—all cross the placenta, disrupt endocrine signaling, increase oxidative stress, and induce epigenetic change, with potential consequences for offspring health [142,143,144,145]. These agents often co-occur in urban environments and may exert additive or synergistic effects on fetal biology, though their cumulative impact on TL remains poorly defined.

PAHs are fused-ring hydrocarbons produced by incomplete combustion. They circulate widely in the atmosphere through transport, deposition, and surface–air exchange [146]. Higher metabolite levels of PAHs in expectant mothers, especially 2-hydroxy-phenanthrene, were linked to shorter telomeres in the infant’s cord-blood cells and to poorer early-life neurobehavior. Mediation analysis suggested the telomere shortening explained much of the PAH-related dip in neurobehavior scores, spotlighting telomere damage as a key biological pathway [147]. Unfortunately, these studies rely on regional exposure estimates rather than individual biomarkers, limiting causal inference.

Phthalates, ubiquitous in plastics and personal-care products, show more complex TL patterns. Gestational exposure elevates maternal oxidative stress, which can damage fetal telomeres because of their guanine content; chronic inflammation provides an additional route [148]. In the EARTH study, five maternal urine samples collected across pregnancy revealed positive links between three metabolites and cord-blood TL: di-isobutyl phthalate (DiBP) in both sexes, mono-n-butyl phthalate (MnBP) in females, and mono-2-ethylhexyl phthalate (MEHP) in males [144]. By contrast, a Chinese birth cohort reported inverse associations between several first-trimester phthalates metabolites and cord-blood TL, suggesting that dose, timing, or specific mixtures may determine directionality [148]. These conflicting results highlight the importance of longitudinal sampling, metabolite-specific effects, and sex-stratified analysis in future phthalate research.

TCS is an antibacterial agent still found in some toothpastes, mouthwashes, soaps, and hand sanitizers. It is absorbed through skin or oral mucosa, reaches peak maternal concentrations 12–18 h after exposure and crosses the placenta [149]. Prenatal TCS exposure has been linked to impaired immune function, pre-term birth, miscarriage risk, low birthweight, and neurodevelopmental delay [150]. Experimental work in gull embryos showed that TCS-induced oxidative stress shortened telomeres in brain cells [151]. Consistently, a human birth-cohort study observed shorter TL in male newborns whose mothers had higher prenatal TCS levels [144]. Although limited in scale, these findings support a sex-specific effect of TCS on fetal telomere biology, potentially mediated by hormonal or epigenetic pathways.

Growing research also connects ambient air pollution with pre-term birth, fetal-growth restriction, increased uterine vascular resistance, impaired placental vascularization, and gestational diabetes, as well as shorter TL in fetal or placental tissues. Preliminary data even suggest that environmental noise may relate to early-onset pre-eclampsia [152]. Table 2 has all the factors that affect offspring telomere length with the relevant studies and evidence.

Despite heterogeneity in study design and pollutant measurement, the convergence of outcomes across multiple cohorts supports environmental contaminants as modifiable risk factors for fetal telomere attrition. Together, these findings place environmental contaminants among the modifiable influences on prenatal telomere biology and long-term offspring health. Future research should prioritize exposure mixtures, critical windows of susceptibility, and standardized telomere assays to improve comparability and mechanistic insights.

## 7. Strength of Evidence Across Maternal Factors

Most associations discussed in this review come from prospective birth cohorts or case–control studies; relatively few stem from randomized or quasi-experimental work. The evidence is strong—that is, replicated in several concordant cohorts with clear biological plausibility—for maternal smoking [124,125,127], high-refined-carbohydrate intake [50,51,52], saturated fat excess [36,54,55], vitamin C and vitamin D sufficiency [41,43,46], adequate folate [47,49], and adherence to a Mediterranean dietary pattern [40,153]. Evidence is moderate—two or more studies with some heterogeneity—for pre-pregnancy obesity [96,98], maternal psychological stress [93,103], n-3:n-6 PUFA balance [2], quality of plant-based diets [69,73], and physical activity when combined with a nutrient-dense diet [34,154]. Evidence remains limited or conflicting—single cohorts, small samples, or inconsistent direction—for caffeine [36,137], triclosan [144], phthalates [144,148], sleep-disordered breathing [115,126], and maternal infection [92]. Across all domains, residual confounding, recall bias in self-reported diet, and limited longitudinal telomere tracking restrict causal inference. Biomarker-based prospective trials with harmonized exposure metrics are needed to clarify dose–response relationships and critical windows of vulnerability.

## 8. Conclusions

Fetal telomere biology is increasingly recognized as a sensitive indicator of the intra-uterine environment and a potential predictor of life-long health. Current evidence shows that favorable maternal behaviors (adequate vitamin C, vitamin D, folate, and magnesium; regular, moderate physical activity paired with a Mediterranean-style diet; and minimal contact with endocrine-disrupting chemicals such as phthalates) align with longer telomeres in cord-blood or placental tissue. Diets rich in fish, nuts, seeds, fruit, vegetables, olives, and legumes and characterized by high total antioxidant capacity demonstrate similar benefits. Conversely, high intake of refined carbohydrates or saturated fat, any alcohol use, prenatal exposure to triclosan, and unmanaged maternal infection are linked to shorter telomeres.

Findings for maternal smoking, sleep quality, polyunsaturated fatty acids, caffeine, psychological stress, overall Mediterranean diet adherence, and maternal obesity remain mixed, with individual studies reporting null, positive, or negative effects. Most available data come from observational cohorts, typically modest in size with heterogeneous exposure assessment, so causal inference is limited and clinical or policy guidance should remain cautious.

Low-risk, biologically plausible actions include encouraging antioxidant-rich eating patterns, ensuring adequate folate and vitamin D, providing effective smoking-cessation support, and offering validated stress-reduction programs; medically supervised moderate exercise is also advisable for its cardiometabolic benefits. Large longitudinal cohorts and intervention trials that incorporate precise exposure metrics, repeated telomere assays, and long-term child follow-up are needed to confirm whether modifying these maternal factors durably influences offspring telomere dynamics and, by extension, chronic disease risk [36,96].

In summary, optimizing maternal nutrition, well-being, and overall lifestyle may foster healthier telomere biology in the next generation, but definitive proof will require trials that address timing, dose, and interactions among multiple prenatal exposures.

## Figures and Tables

**Table 1:** Physical activity recommendations for pregnant women by various organizations.

Organization	Recommended Duration of Physical Activity
American College of Obstetricians and Gynecologists (ACOG) [84]	150 min of moderate-intensity aerobic exercise each week
U.S. Department of Health and Human Services [85]	150 min of moderate-intensity aerobic exercise each week
Royal Australian and New Zealand College of Obstetricians and Gynecologists (RAZCOG) [86]	150–300 min of regular exercise weekly or 70–150 min of vigorous exercise weekly (with sessions not exceeding 60 min)
Society of Obstetricians and Gynecologists of Canada (SOGC) [87]	No official exercise duration recommendation

**Table 2:** Maternal factors associated with longer or shorter telomere length in offspring.

Maternal Factor	Association with Offspring’s TL	Key Studies *
Vitamin C intake	+	[41,43]
Vitamin D status	+	[36,46]
Folate intake	+	[47,49,133]
Magnesium	±	[11]
Mediterranean-style diet (MD)	+ (female), ± (male)	[40,64,153]
High-antioxidant foods	+	[51]
Refined carbohydrates/high glycemic load	–	[50,51,52]
Saturated fat intake	–	[36,54,55]
Alcohol consumption	–	[127,132]
Caffeine intake	+	[36,137]
Physical activity + MD	+	[34]
Maternal pre-pregnancy BMI	–	[96,98]
Adequate gestational weight gain	+	[91]
Maternal stress	–/±	[93,103,111]
Poor sleep/sleep-disordered breathing	–/±	[111,115,116]
Smoking (active)	–/+	[124,125,126,127,128]; outlier [129]
Second-hand smoke	–	[124]
Triclosan exposure	– (male-specific)	[144,151]
Phthalate exposure	±	[144,148]
PAH exposure	–	[147]
Maternal infection (bacterial or viral)	–	[94,118]

*, not exhaustive, illustrates most-cited evidence; +, positive; −, negative; ±, mixed or inconclusive.

## Data Availability

Not applicable.

## Abbreviations

The following abbreviations are used in this manuscript:

PA	Physical activity
PAHs	Polycyclic aromatic hydrocarbons
MD	Mediterranean diet
TL	Telomere length
ROS	Reactive oxygen species
SSBs	Sugar-sweetened beverages
PTB	Pre-term birth
BMI	Body mass index
CO	Carbon monoxide
COX	Cytochrome-c oxidase
IPF	Idiopathic pulmonary fibrosis
COPD	Chronic obstructive pulmonary disease
HPA	Hypothalamic–pituitary–adrenal
ACTH	Adrenocorticotropic hormone
ADHD	Attention-deficit/hyperactivity disorder
CRH	Corticotropin-releasing hormone
MDD	Major Depressive Disorder
Q-PCR	Quantitative polymerase chain reaction
ACOG	American College of Obstetricians and Gynecologists
RANZCOG	Royal Australian and New Zealand College of Obstetricians and Gynecologists
SOGC	Society of Obstetricians and Gynecologists of Canada
ADA	American Dietetic Association
PUFA	Polyunsaturated fatty acid
TAC	Total antioxidant capacity
WHO	World Health Organization

## References

[1] Moyzis R.K., Buckingham J.M., Cram L.S., Dani M., Deaven L.L., Jones M.D., Meyne J., Ratliff R.L., Wu J.-R. (1988). A highly conserved repetitive DNA sequence, (TTAGGG)n, present at the telomeres of human chromosomes. Proc. Natl. Acad. Sci. USA.

[2] Gorenjak V., Petrelis A.M., Stathopoulou M.G., Visvikis-Siest S. (2020). Telomere length determinants in childhood. Clin. Chem. Lab. Med..

[3] Sanders J.L., Newman A.B. (2013). Telomere length in epidemiology: A biomarker of aging, age-related disease, both, or neither?. Epidemiol. Rev..

[4] Entringer S., de Punder K., Buss C., Wadhwa P.D. (2018). The fetal programming of telomere biology hypothesis: An update. Philos. Trans. R. Soc. B Biol. Sci..

[5] Yu H.J., Byun Y.H., Park C.-K. (2024). Techniques for assessing telomere length: A methodological review. Comput. Struct. Biotechnol. J..

[6] Wang Y., Savage S.A., Alsaggaf R., Aubert G., Dagnall C.L., Spellman S.R., Lee S.J., Hicks B., Jones K., Katki H.A. (2018). Telomere length calibration from qPCR measurement: Limitations of current method. Cells.

[7] De Meyer T., Rietzschel E.R., De Buyzere M.L., De Bacquer D., Van Criekinge W., De Backer G.G., Gillebert T.C., Van Oostveldt P., Bekaert S. (2007). Paternal age at birth is an important determinant of offspring telomere length. Hum. Mol. Genet..

[8] Vaiserman A., Krasnienkov D. (2020). Telomere Length as a Marker of Biological Age: State-of-the-Art, Open Issues, and Future Perspectives. Front. Genet..

[9] Montemurro T., Lavazza C., Montelatici E., Budelli S., La Rosa S., Barilani M., Mei C., Manzini P., Ratti I., Cimoni S. (2024). Off-the-Shelf Cord-Blood Mesenchymal Stromal Cells: Production, Quality Control, and Clinical Use. Cells.

[10] Talwadekar M.D., Kale V.P., Limaye L.S. (2015). Placenta-derived mesenchymal stem cells possess better immunoregulatory properties compared to their cord-derived counterparts-a paired sample study. Sci. Rep..

[11] Vahter M., Broberg K., Harari F. (2020). Placental and Cord Blood Telomere Length in Relation to Maternal Nutritional Status. J. Nutr..

[12] Gluckman P.D., Hanson M.A., Cooper C., Thornburg K.L. (2008). Effect of in utero and early-life conditions on adult health and disease. N. Engl. J. Med..

[13] Chen L., Tan K.M.L., Gong M., Chong M.F.F., Tan K.H., Chong Y.S., Meaney M.J., Gluckman P.D., Eriksson J.G., Karnani N. (2022). Variability in newborn telomere length is explained by inheritance and intrauterine environment. BMC Med..

[14] Entringer S., Buss C., Wadhwa P.D. (2012). Prenatal stress, telomere biology, and fetal programming of health and disease risk. Sci. Signal..

[15] Entringer S., Buss C., Wadhwa P.D. (2010). Prenatal stress and developmental programming of human health and disease risk: Concepts and integration of empirical findings. Curr. Opin. Endocrinol. Diabetes Obes..

[16] Martens D.S., Van Der Stukken C., Derom C., Thiery E., Bijnens E.M., Nawrot T.S. (2021). Newborn telomere length predicts later life telomere length: Tracking telomere length from birth to child- and adulthood. eBioMedicine.

[17] Okuda K., Bardeguez A., Gardner J.P., Rodriguez P., Ganesh V., Kimura M., Skurnick J., Awad G., Aviv A. (2002). Telomere length in the newborn. Pediatr. Res..

[18] Abu-Awwad S.-A., Craina M., Gluhovschi A., Ciordas P.D., Marian C., Boscu L., Bernad E., Iurciuc M., Abu-Awwad A., Iurciuc S. (2023). Linking Pregnancy and Long-Term Health: The Impact of Cardiovascular Risk on Telomere Shortening in Pregnant Women. Medicina.

[19] Levstek T., Kozjek E., Dolžan V., Trebušak Podkrajšek K. (2020). Telomere Attrition in Neurodegenerative Disorders. Front. Cell. Neurosci..

[20] Zheng B., Fu J. (2023). Telomere dysfunction in some pediatric congenital and growth-related diseases. Front. Pediatr..

[21] Latifovic L., Peacock S.D., Massey T.E., King W.D. (2016). The Influence of Alcohol Consumption, Cigarette Smoking, and Physical Activity on Leukocyte Telomere Length. Cancer Epidemiol. Biomark. Prev..

[22] Tsatsakis A., Oikonomopoulou T., Nikolouzakis T.K., Vakonaki E., Tzatzarakis M., Flamourakis M., Renieri E., Fragkiadaki P., Iliaki E., Bachlitzanaki M. (2023). Role of telomere length in human carcinogenesis. Int. J. Oncol..

[23] Baliou S., Pelagiadis I., Apetroaei M.-M., Vakonaki E., Arsene A.L., Hatzidaki E., Tzatzarakis M.N., Ioannou P., Tsatsakis A., Stiakaki E. (2025). The Telomere Length Signature in Leukemias—From Molecular Mechanisms Underlying Telomere Shortening to Immunotherapeutic Options Against Telomerase. Cancers.

[24] Barker D. (1993). Fetal origins of coronary heart disease. Br. Heart J..

[25] Lowensohn R.I., Stadler D.D., Naze C. (2016). Current Concepts of Maternal Nutrition. Obstet. Gynecol. Surv..

[26] Bischoff C., Graakjaer J., Petersen H.C., Hjelmborg J., Vaupel J.W., Bohr V., Koelvraa S., Christensen K. (2005). The heritability of telomere length among the elderly and oldest-old. Twin Res. Hum. Genet..

[27] Vasa-Nicotera M., Brouilette S., Mangino M., Thompson J.R., Braund P., Clemitson J.R., Mason A., Bodycote C.L., Raleigh S.M., Louis E. (2005). Mapping of a major locus that determines telomere length in humans. Am. J. Hum. Genet..

[28] Andrew T., Aviv A., Falchi M., Surdulescu G.L., Gardner J.P., Lu X., Kimura M., Kato B.S., Valdes A.M., Spector T.D. (2006). Mapping genetic loci that determine leukocyte telomere length in a large sample of unselected female sibling pairs. Am. J. Hum. Genet..

[29] Cesare A.J., Reddel R.R. (2010). Alternative lengthening of telomeres: Models, mechanisms and implications. Nat. Rev. Genet..

[30] Broer L., Codd V., Nyholt D.R., Deelen J., Mangino M., Willemsen G., Albrecht E., Amin N., Beekman M., De Geus E.J. (2013). Meta-analysis of telomere length in 19 713 subjects reveals high heritability, stronger maternal inheritance and a paternal age effect. Eur. J. Hum. Genet..

[31] Nawrot T.S., Staessen J.A., Gardner J.P., Aviv A. (2004). Telomere length and possible link to X chromosome. Lancet.

[32] Fagan E., Sun F., Bae H., Elo I., Andersen S.L., Lee J., Christensen K., Thyagarajan B., Sebastiani P., Perls T. (2017). Telomere length is longer in women with late maternal age. Menopause.

[33] Ye Q., Apsley A.T., Etzel L., Hastings W.J., Kozlosky J.T., Walker C., Wolf S.E., Shalev I. (2023). Telomere length and chronological age across the human lifespan: A systematic review and meta-analysis of 414 study samples including 743,019 individuals. Ageing Res. Rev..

[34] Flor-Alemany M., Acosta-Manzano P., Migueles J.H., Varela-López A., Baena-García L., Quiles J.L., Aparicio V.A. (2023). Influence of an exercise intervention plus an optimal Mediterranean diet adherence during pregnancy on the telomere length of the placenta. The GESTAFIT project. Placenta.

[35] Moshfeghinia R., Torabi A., Mostafavi S., Rahbar S., Moradi M.S., Sadeghi E., Mootz J., Vardanjani H.M. (2023). Maternal psychological stress during pregnancy and newborn telomere length: A systematic review and meta-analysis. BMC Psychiatry.

[36] Habibi N., Bianco-Miotto T., Phoi Y.Y., Jankovic-Karasoulos T., Roberts C.T., Grieger J.A. (2021). Maternal diet and offspring telomere length: A systematic review. Nutr. Rev..

[37] Baliou S., Ioannou P., Apetroaei M.-M., Vakonaki E., Fragkiadaki P., Kirithras E., Tzatzarakis M.N., Arsene A.L., Docea A.O., Tsatsakis A. (2024). The Impact of the Mediterranean Diet on Telomere Biology: Implications for Disease Management—A Narrative Review. Nutrients.

[38] Herlin M., Broberg K., Igra A.M., Li H., Harari F., Vahter M. (2019). Exploring telomere length in mother-newborn pairs in relation to exposure to multiple toxic metals and potential modifying effects by nutritional factors. BMC Med..

[39] Andreu-Sánchez S., Aubert G., Ripoll-Cladellas A., Henkelman S., Zhernakova D.V., Sinha T., Kurilshikov A., Cenit M.C., Jan Bonder M., Franke L. (2022). Genetic, parental and lifestyle factors influence telomere length. Commun. Biol..

[40] Galiè S., Canudas S., Muralidharan J., García-Gavilán J., Bulló M., Salas-Salvadó J. (2020). Impact of Nutrition on Telomere Health: Systematic Review of Observational Cohort Studies and Randomized Clinical Trials. Adv. Nutr..

[41] Myers K.O., Boubakari I., Yusuf K.K., Mauck D.E., Salihu H.M. (2021). The effect of maternal vitamin C intake on fetal telomere length. J. Matern. Fetal Neonatal Med..

[42] Rumbold A., Ota E., Nagata C., Shahrook S., Crowther C.A. (2015). Vitamin C supplementation in pregnancy. Cochrane Database Syst. Rev..

[43] Cai Y., Zhong Y.D., Zhang H., Lu P.L., Liang Y.Y., Hu B., Wu H. (2023). Association between dietary vitamin C and telomere length: A cross-sectional study. Front. Nutr..

[44] Marcon F., Siniscalchi E., Crebelli R., Saieva C., Sera F., Fortini P., Simonelli V., Palli D. (2012). Diet-related telomere shortening and chromosome stability. Mutagenesis.

[45] Wagner C.L., Taylor S.N., Johnson D.D., Hollis B.W. (2012). The role of vitamin D in pregnancy and lactation: Emerging concepts. Women’s Health.

[46] Kim J.H., Kim G.J., Lee D., Ko J.H., Lim I., Bang H., Koes B.W., Seong B., Lee D.C. (2018). Higher maternal vitamin D concentrations are associated with longer leukocyte telomeres in newborns. Matern. Child Nutr..

[47] Entringer S., Epel E.S., Lin J., Blackburn E.H., Buss C., Shahbaba B., Gillen D.L., Venkataramanan R., Simhan H.N., Wadhwa P.D. (2015). Maternal Folate Concentration in Early Pregnancy and Newborn Telomere Length. Ann. Nutr. Metab..

[48] Magnano San Lio R., Maugeri A., La Rosa M.C., Giunta G., Panella M., Cianci A., Caruso M.A.T., Agodi A., Barchitta M. (2022). Nutrient intakes and telomere length of cell-free circulating DNA from amniotic fluid: Findings from the Mamma & Bambino cohort. Sci. Rep..

[49] Zhou D., Li Z., Sun Y., Yan J., Huang G., Li W. (2022). Early Life Stage Folic Acid Deficiency Delays the Neurobehavioral Development and Cognitive Function of Rat Offspring by Hindering De Novo Telomere Synthesis. Int. J. Mol. Sci..

[50] Leung C.W., Laraia B.A., Needham B.L., Rehkopf D.H., Adler N.E., Lin J., Blackburn E.H., Epel E.S. (2014). Soda and cell aging: Associations between sugar-sweetened beverage consumption and leukocyte telomere length in healthy adults from the National Health and Nutrition Examination Surveys. Am. J. Public Health.

[51] García-Calzón S., Moleres A., Martínez-González M.A., Martínez J.A., Zalba G., Marti A. (2015). Dietary total antioxidant capacity is associated with leukocyte telomere length in a children and adolescent population. Clin. Nutr..

[52] Salihu H.M., Adegoke K.K., King L.M., Daas R., Paothong A., Pradhan A., Aliyu M.H., Whiteman V.E. (2018). Effects of Maternal Carbohydrate and Fat Intake on Fetal Telomere Length. South. Med. J..

[53] Sweeting A., Mijatovic J., Brinkworth G.D., Markovic T.P., Ross G.P., Brand-Miller J., Hernandez T.L. (2021). The carbohydrate threshold in pregnancy and gestational diabetes: How low can we go?. Nutrients.

[54] Tucker L.A. (2019). Milk Fat Intake and Telomere Length in U.S. Women and Men: The Role of the Milk Fat Fraction. Oxidative Med. Cell. Longev..

[55] Song Y., You N.C., Song Y., Kang M.K., Hou L., Wallace R., Eaton C.B., Tinker L.F., Liu S. (2013). Intake of small-to-medium-chain saturated fatty acids is associated with peripheral leukocyte telomere length in postmenopausal women. J. Nutr..

[56] Xiong T., Wu Y., Huang L., Chen X., Zhang Y., Zhong C., Gao Q., Hong M., Hu X., Yang X. (2021). Association between the maternal protein nutrition status during pregnancy and the risk of preterm birth. Matern. Child Nutr..

[57] Switkowski K.M., Jacques P.F., Must A., Kleinman K.P., Gillman M.W., Oken E. (2016). Maternal protein intake during pregnancy and linear growth in the offspring. Am. J. Clin. Nutr..

[58] Kasielski M., Eusebio M.O., Pietruczuk M., Nowak D. (2016). The relationship between peripheral blood mononuclear cells telomere length and diet—unexpected effect of red meat. Nutr. J..

[59] Lis N., Lamnisos D., Bograkou-Tzanetakou A., Hadjimbei E., Tzanetakou I.P. (2023). Preterm Birth and Its Association with Maternal Diet, and Placental and Neonatal Telomere Length. Nutrients.

[60] Blumfield M.L., Collins C.E. (2014). High-protein diets during pregnancy: healthful or harmful for offspring?. Am. J. Clin. Nutr..

[61] Zaragoza-Martí A., Ruiz-Ródenas N., Herranz-Chofre I., Sánchez-SanSegundo M., Serrano Delgado V.C., Hurtado-Sánchez J.A. (2022). Adherence to the Mediterranean Diet in Pregnancy and Its Benefits on Maternal-Fetal Health: A Systematic Review of the Literature. Front. Nutr..

[62] Assaf-Balut C., García de la Torre N., Fuentes M., Durán A., Bordiú E., Del Valle L., Valerio J., Jiménez I., Herraiz M.A., Izquierdo N. (2018). A high adherence to six food targets of the mediterranean diet in the late first trimester is associated with a reduction in the risk of materno-foetal outcomes: The St. Carlos gestational diabetes mellitus prevention study. Nutrients.

[63] Boccardi V., Esposito A., Rizzo M.R., Marfella R., Barbieri M., Paolisso G. (2013). Mediterranean diet, telomere maintenance and health status among elderly. PLoS ONE.

[64] García-Calzón S., Martínez-González M.A., Razquin C., Arós F., Lapetra J., Martínez J.A., Zalba G., Marti A. (2016). Mediterranean diet and telomere length in high cardiovascular risk subjects from the PREDIMED-NAVARRA study. Clin. Nutr..

[65] Meinilä J., Perälä M.M., Kautiainen H., Männistö S., Kanerva N., Shivappa N., Hébert J.R., Iozzo P., Guzzardi M.A., Eriksson J.G. (2019). Healthy diets and telomere length and attrition during a 10-year follow-up. Eur. J. Clin. Nutr..

[66] Sebastiani G., Herranz Barbero A., Borrás-Novell C., Alsina Casanova M., Aldecoa-Bilbao V., Andreu-Fernández V., Pascual Tutusaus M., Ferrero Martínez S., Gómez Roig M.D., García-Algar O. (2019). The Effects of Vegetarian and Vegan Diet during Pregnancy on the Health of Mothers and Offspring. Nutrients.

[67] Agnoli C., Baroni L., Bertini I., Ciappellano S., Fabbri A., Papa M., Pellegrini N., Sbarbati R., Scarino M.L., Siani V. (2017). Position paper on vegetarian diets from the working group of the Italian Society of Human Nutrition. Nutr. Metab. Cardiovasc. Dis..

[68] Dwaraka V.B., Aronica L., Carreras-Gallo N., Robinson J.L., Hennings T., Carter M.M., Corley M.J., Lin A., Turner L., Smith R. (2024). Unveiling the epigenetic impact of vegan vs. omnivorous diets on aging: Insights from the Twins Nutrition Study (TwiNS). BMC Med..

[69] Tucker L.A. (2021). Fruit and Vegetable Intake and Telomere Length in a Random Sample of 5448 U.S. Adults. Nutrients.

[70] Tucker L.A. (2018). Dietary Fiber and Telomere Length in 5674 U.S. Adults: An NHANES Study of Biological Aging. Nutrients.

[71] Bountziouka V., Nelson C.P., Wang Q., Musicha C., Codd V., Samani N.J. (2023). Dietary Patterns and Practices and Leucocyte Telomere Length: Findings from the UK Biobank. J. Acad. Nutr. Diet..

[72] Cinegaglia N., Antoniazzi L., Rosa D., Miranda D., Acosta-Navarro J., Bortolotto L., Hong V., Sandrim V. (2019). Shortening telomere is associated with subclinical atherosclerosis biomarker in omnivorous but not in vegetarian healthy men. Aging.

[73] Li X., Li M., Cheng J., Guan S., Hou L., Zu S., Yang L., Wu H., Li H., Fan Y. (2024). Association of healthy and unhealthy plant-based diets with telomere length. Clin. Nutr..

[74] Craig W.J., Mangels A.R. (2009). Position of the American Dietetic Association: Vegetarian diets. J. Am. Diet. Assoc..

[75] Richter M., Boeing H., Grünewald-Funk D., Heseker H., Kroke A., Leschik-Bonnet E., Oberritter H., Strohm D., Watzl B. (2016). Vegan diet. Position of the German nutrition society (DGE). Ernahr. Umsch..

[76] Bauer I., Hartkopf J., Kullmann S., Schleger F., Hallschmid M., Pauluschke-Fröhlich J., Fritsche A., Preissl H. (2020). Spotlight on the fetus: How physical activity during pregnancy influences fetal health: A narrative review. BMJ Open Sport Exerc. Med..

[77] May L.E., Allen J.J., Gustafson K.M. (2016). Fetal and maternal cardiac responses to physical activity and exercise during pregnancy. Early Hum. Dev..

[78] Clapp J.F., Kim H., Burciu B., Lopez B. (2000). Beginning regular exercise in early pregnancy: Effect on fetoplacental growth. Am. J. Obstet. Gynecol..

[79] Gomez-Cabrera M.C., Domenech E., Viña J. (2008). Moderate exercise is an antioxidant: Upregulation of antioxidant genes by training. Free. Radic. Biol. Med..

[80] Akay G.G. (2022). Telomeres and Psychological Stress: Perspective on Psychopathologies. Noro Psikiyatr. Ars..

[81] De Lange T. (2018). Shelterin-mediated telomere protection. Annu. Rev. Genet..

[82] Semeraro M.D., Smith C., Kaiser M., Levinger I., Duque G., Gruber H.-J., Herrmann M. (2020). Physical activity, a modulator of aging through effects on telomere biology. Aging.

[83] Pérez L.M., Amaral M.A., Mundstock E., Barbé-Tuana F.M., Guma F., Jones M.H., Machado D.C., Sarria E.E., Marques E.M.M., Preto L.T. (2017). Effects of Diet on Telomere Length: Systematic Review and Meta-Analysis. Public Health Genom..

[84] (2020). Physical Activity and Exercise During Pregnancy and the Postpartum Period: ACOG Committee Opinion, Number 804. Obstet. Gynecol..

[85] Piercy K.L., Troiano R.P., Ballard R.M., Carlson S.A., Fulton J.E., Galuska D.A., George S.M., Olson R.D. (2018). The Physical Activity Guidelines for Americans. Jama.

[86] Brown W.J., Hayman M., Haakstad L.A., Lamerton T., Mena G.P., Green A., Keating S.E., Gomes G.A., Coombes J.S., Mielke G.I. (2022). Australian guidelines for physical activity in pregnancy and postpartum. J. Sci. Med. Sport.

[87] Mottola M.F., Davenport M.H., Ruchat S.M., Davies G.A., Poitras V.J., Gray C.E., Jaramillo Garcia A., Barrowman N., Adamo K.B., Duggan M. (2018). 2019 Canadian guideline for physical activity throughout pregnancy. Br. J. Sports Med..

[88] Berghella V., Saccone G. (2017). Exerc. Pregnancy!. Am. J. Obstet. Gynecol..

[89] Hinman S.K., Smith K.B., Quillen D.M., Smith M.S. (2015). Exercise in Pregnancy: A Clinical Review. Sports Health.

[90] Davenport M.H., Ruchat S.M., Poitras V.J., Jaramillo Garcia A., Gray C.E., Barrowman N., Skow R.J., Meah V.L., Riske L., Sobierajski F. (2018). Prenatal exercise for the prevention of gestational diabetes mellitus and hypertensive disorders of pregnancy: A systematic review and meta-analysis. Br. J. Sports Med..

[91] Tsakiridis I., Bakaloudi D.R., Oikonomidou A.C., Dagklis T., Chourdakis M. (2020). Exercise during pregnancy: A comparative review of guidelines. J. Perinat. Med..

[92] Lazarides C., Epel E.S., Lin J., Blackburn E.H., Voelkle M.C., Buss C., Simhan H.N., Wadhwa P.D., Entringer S. (2019). Maternal pro-inflammatory state during pregnancy and newborn leukocyte telomere length: A prospective investigation. Brain Behav. Immun..

[93] Send T.S., Gilles M., Codd V., Wolf I., Bardtke S., Streit F., Strohmaier J., Frank J., Schendel D., Sütterlin M.W. (2017). Telomere length in newborns is related to maternal stress during pregnancy. Neuropsychopharmacology.

[94] Tung K.T.S., Hung C.M.W., Chan K.L., Wong R.S., Tsang H.W., Wong W.H.S., Lo C.K.M., Tso W.W.Y., Chua G.T., Yee B.K. (2021). Influence of Maternal Infection and Pregnancy Complications on Cord Blood Telomere Length. Oxidative Med. Cell. Longev..

[95] Leddy M.A., Power M.L., Schulkin J. (2008). The impact of maternal obesity on maternal and fetal health. Rev. Obstet. Gynecol..

[96] Martens D.S., Plusquin M., Gyselaers W., De Vivo I., Nawrot T.S. (2016). Maternal pre-pregnancy body mass index and newborn telomere length. BMC Med..

[97] Whitaker R.C. (2004). Predicting preschooler obesity at birth: The role of maternal obesity in early pregnancy. Pediatrics.

[98] Wei B., Shao Y., Liang J., Tang P., Mo M., Liu B., Huang H., Tan H.J.J., Huang D., Liu S. (2021). Maternal overweight but not paternal overweight before pregnancy is associated with shorter newborn telomere length: Evidence from Guangxi Zhuang birth cohort in China. BMC Pregnancy Childbirth.

[99] Maugeri A., Magnano San Lio R., La Rosa M.C., Giunta G., Panella M., Cianci A., Caruso M.A.T., Agodi A., Barchitta M. (2022). The Relationship between Telomere Length and Gestational Weight Gain: Findings from the Mamma & Bambino Cohort. Biomedicines.

[100] Reyes F.I., Boroditsky R.S., Winter J.S., Faiman C. (1974). Studies on human sexual development. II. Fetal Matern. Serum Gonadotropin Sex Steroid concentrations. J. Clin. Endocrinol. Metab..

[101] Vina J., Gambini J., Lopez-Grueso R., Abdelaziz K.M., Jove M., Borras C. (2011). Females live longer than males: Role of oxidative stress. Curr. Pharm. Des..

[102] Dolatian M., Sharifi N., Mahmoodi Z., Fathnezhad-kazemi A., Bahrami-vazir E., Rashidian T. (2020). Weight gain during pregnancy and its associated factors: A Path analysis. Nurs. Open.

[103] Entringer S., Epel E.S., Lin J., Buss C., Shahbaba B., Blackburn E.H., Simhan H.N., Wadhwa P.D. (2013). Maternal psychosocial stress during pregnancy is associated with newborn leukocyte telomere length. Am. J. Obstet. Gynecol..

[104] Mayer S.E., Guan J., Lin J., Hamlat E., Parker J.E., Brownell K., Price C., Mujahid M., Tomiyama A.J., Slavich G.M. (2023). Intergenerational effects of maternal lifetime stressor exposure on offspring telomere length in Black and White women. Psychol. Med..

[105] Leistner C., Menke A. (2020). Hypothalamic-pituitary-adrenal axis and stress. Handb. Clin. Neurol..

[106] Lin J., Epel E. (2022). Stress and telomere shortening: Insights from cellular mechanisms. Ageing Res. Rev..

[107] Salihu H.M., King L.M., Nwoga C., Paothong A., Pradhan A., Marty P.J., Daas R., Whiteman V.E. (2016). Association Between Maternal-Perceived Psychological Stress and Fetal Telomere Length. South. Med. J..

[108] Dowell J., Elser B.A., Schroeder R.E., Stevens H.E. (2019). Cellular stress mechanisms of prenatal maternal stress: Heat shock factors and oxidative stress. Neurosci. Lett..

[109] Marchetto N.M., Glynn R.A., Ferry M.L., Ostojic M., Wolff S.M., Yao R., Haussmann M.F. (2016). Prenatal stress and newborn telomere length. Am. J. Obstet. Gynecol..

[110] Verner G., Epel E., Lahti-Pulkkinen M., Kajantie E., Buss C., Lin J., Blackburn E., Räikkönen K., Wadhwa P.D., Entringer S. (2021). Maternal Psychological Resilience During Pregnancy and Newborn Telomere Length: A Prospective Study. Am. J. Psychiatry.

[111] Ämmälä A.-J., Vitikainen E.I.K., Hovatta I., Paavonen J., Saarenpää-Heikkilä O., Kylliäinen A., Pölkki P., Porkka-Heiskanen T., Paunio T. (2020). Maternal stress or sleep during pregnancy are not reflected on telomere length of newborns. Sci. Rep..

[112] Bosquet Enlow M., Petty C.R., Hacker M.R., Burris H.H. (2021). Maternal psychosocial functioning, obstetric health history, and newborn telomere length. Psychoneuroendocrinology.

[113] Izano M.A., Cushing L.J., Lin J., Eick S.M., Goin D.E., Epel E., Woodruff T.J., Morello-Frosch R. (2020). The association of maternal psychosocial stress with newborn telomere length. PLoS ONE.

[114] Sedov I.D., Cameron E.E., Madigan S., Tomfohr-Madsen L.M. (2018). Sleep quality during pregnancy: A meta-analysis. Sleep Med. Rev..

[115] Liu Q., Song L., Fan G., Wu M., Bi J., Xu L., Xiong C., Xia W., Cao Z., Xu S. (2023). Associations of self-reported sleep duration and sleep quality during pregnancy with newborn telomere length. Sleep Health.

[116] Salihu H.M., King L., Patel P., Paothong A., Pradhan A., Louis J., Naik E., Marty P.J., Whiteman V. (2015). Association between maternal symptoms of sleep disordered breathing and fetal telomere length. Sleep.

[117] Auriti C., De Rose D.U., Santisi A., Martini L., Piersigilli F., Bersani I., Ronchetti M.P., Caforio L. (2021). Pregnancy and viral infections: Mechanisms of fetal damage, diagnosis and prevention of neonatal adverse outcomes from cytomegalovirus to SARS-CoV-2 and Zika virus. Biochim. Et Biophys. Acta Mol. Basis Dis..

[118] Liu B., Maekawa T., Chatton B., Ishii S. (2016). In utero TNF-α treatment induces telomere shortening in young adult mice in an ATF7-dependent manner. FEBS Open Bio..

[119] Maekawa T., Liu B., Nakai D., Yoshida K., Nakamura K.-i., Yasukawa M., Koike M., Takubo K., Chatton B., Ishikawa F. (2018). ATF7 mediates TNF-α–induced telomere shortening. Nucleic Acids Res..

[120] Banerjee S., Deacon A., Suter M.A., Aagaard K.M. (2022). Understanding the Placental Biology of Tobacco Smoke, Nicotine, and Marijuana (THC) Exposures During Pregnancy. Clin. Obstet. Gynecol..

[121] Garrabou G., Hernàndez A.S., Catalán García M., Morén C., Tobías E., Córdoba S., López M., Figueras F., Grau J.M., Cardellach F. (2016). Molecular basis of reduced birth weight in smoking pregnant women: Mitochondrial dysfunction and apoptosis. Addict. Biol..

[122] Chełchowska M., Gajewska J., Ambroszkiewicz J., Mazur J., Ołtarzewski M., Maciejewski T.M. (2021). Influence of Oxidative Stress Generated by Smoking during Pregnancy on Glutathione Status in Mother-Newborn Pairs. Antioxidants.

[123] Valdes A.M., Andrew T., Gardner J.P., Kimura M., Oelsner E., Cherkas L.F., Aviv A., Spector T.D. (2005). Obesity, cigarette smoking, and telomere length in women. Lancet.

[124] Liu B., Song L., Zhang L., Wu M., Wang L., Cao Z., Xiong C., Zhang B., Li Y., Xia W. (2020). Prenatal second-hand smoke exposure and newborn telomere length. Pediatr. Res..

[125] Mirzakhani H., De Vivo I., Leeder J.S., Gaedigk R., Vyhlidal C.A., Weiss S.T., Tantisira K. (2017). Early pregnancy intrauterine fetal exposure to maternal smoking and impact on fetal telomere length. Eur. J. Obstet. Gynecol. Reprod. Biol..

[126] Salihu H.M., Pradhan A., King L., Paothong A., Nwoga C., Marty P.J., Whiteman V. (2015). Impact of intrauterine tobacco exposure on fetal telomere length. Am. J. Obstet. Gynecol..

[127] Ip P., Chung B.H., Ho F.K., Chan G.C., Deng W., Wong W.H., Lee S.L., Chan P.Y., Ying D., Wong W.L. (2017). Prenatal Tobacco Exposure Shortens Telomere Length in Children. Nicotine Tob. Res..

[128] Theall K.P., McKasson S., Mabile E., Dunaway L.F., Drury S.S. (2013). Early hits and long-term consequences: Tracking the lasting impact of prenatal smoke exposure on telomere length in children. Am. J. Public Health.

[129] Almanzar G., Eberle G., Lassacher A., Specht C., Koppelstaetter C., Heinz-Erian P., Trawöger R., Bernhard D., Prelog M. (2013). Maternal cigarette smoking and its effect on neonatal lymphocyte subpopulations and replication. BMC Pediatr..

[130] Wadhwa P.D., Buss C., Entringer S., Swanson J.M. (2009). Developmental origins of health and disease: Brief history of the approach and current focus on epigenetic mechanisms. Semin. Reprod. Med..

[131] Howell M.P., Jones C.W., Herman C.A., Mayne C.V., Fernandez C., Theall K.P., Esteves K.C., Drury S.S. (2022). Impact of prenatal tobacco smoking on infant telomere length trajectory and ADHD symptoms at 18 months: A longitudinal cohort study. BMC Med..

[132] Maugeri A., Barchitta M., Magnano San Lio R., La Rosa M.C., La Mastra C., Favara G., Ferlito M., Giunta G., Panella M., Cianci A. (2021). The effect of alcohol on telomere length: A systematic review of epidemiological evidence and a pilot study during pregnancy. Int. J. Environ. Res. Public Health.

[133] Topiwala A., Taschler B., Ebmeier K.P., Smith S., Zhou H., Levey D.F., Codd V., Samani N.J., Gelernter J., Nichols T.E. (2022). Alcohol consumption and telomere length: Mendelian randomization clarifies alcohol’s effects. Mol. Psychiatry.

[134] Schölin L., Watson J., Dyson J., Smith L.A. (2019). Alcohol Guidelines for Pregnant Women: Barriers and Enablers for Midwives to Deliver Advice.

[135] Lui S., Jones R.L., Robinson N.J., Greenwood S.L., Aplin J.D., Tower C.L. (2014). Detrimental effects of ethanol and its metabolite acetaldehyde, on first trimester human placental cell turnover and function. PLoS ONE.

[136] Harpaz T., Abumock H., Beery E., Edel Y., Lahav M., Rozovski U., Uziel O. (2018). The Effect of Ethanol on Telomere Dynamics and Regulation in Human Cells. Cells.

[137] Griffin I., Ibrahimou B., Navejar N., Aggarwal A., Myers K., Mauck D., Yusuf K.K., Wudil U.J., Aliyu M.H., Salihu H.M. (2020). Maternal Caffeine Consumption and Racial Disparities in Fetal Telomere Length. Int. J. MCH AIDS.

[138] Knutti R., Rothweiler H., Schlatter C. (1981). Effect of pregnancy on the pharmacokinetics of caffeine. Eur. J. Clin. Pharmacol..

[139] Rhee J., Kim R., Kim Y., Tam M., Lai Y., Keum N., Oldenburg C.E. (2015). Maternal Caffeine Consumption during Pregnancy and Risk of Low Birth Weight: A Dose-Response Meta-Analysis of Observational Studies. PLoS ONE.

[140] World Health Organization (2016). WHO Recommendations on Antenatal Care for a Positive Pregnancy Experience.

[141] Gynecologists, ACoOa (2010). ACOG CommitteeOpinion No. 462: Moderate caffeine consumption during pregnancy. Obstet. Gynecol..

[142] Hlisníková H., Petrovičová I., Kolena B., Šidlovská M., Sirotkin A. (2020). Effects and Mechanisms of Phthalates’ Action on Reproductive Processes and Reproductive Health: A Literature Review. Int. J. Environ. Res. Public Health.

[143] Drwal E., Rak A., Gregoraszczuk E.L. (2019). Review: Polycyclic aromatic hydrocarbons (PAHs)-Action on placental function and health risks in future life of newborns. Toxicology.

[144] Michels K.B., De Vivo I., Calafat A.M., Binder A.M. (2020). In utero exposure to endocrine-disrupting chemicals and telomere length at birth. Environ. Res..

[145] Khoshhali M., Amin M.M., Fatehizadeh A., Ebrahimi A., Taheri E., Kelishadi R. (2020). Impact of prenatal triclosan exposure on gestational age and anthropometric measures at birth: A systematic review and meta-analysis. J. Res. Med. Sci..

[146] Zhang Y., Dong S., Wang H., Tao S., Kiyama R. (2016). Biological impact of environmental polycyclic aromatic hydrocarbons (ePAHs) as endocrine disruptors. Environ. Pollut..

[147] Nie J., Li J., Cheng L., Deng Y., Li Y., Yan Z., Duan L., Niu Q., Tang D. (2019). Prenatal polycyclic aromatic hydrocarbons metabolites, cord blood telomere length, and neonatal neurobehavioral development. Environ. Res..

[148] Song L., Liu B., Wu M., Zhang L., Wang L., Zhang B., Xiong C., Li Y., Cao Z., Wang Y. (2019). Prenatal Exposure to Phthalates and Newborn Telomere Length: A Birth Cohort Study in Wuhan, China. Environ. Health Perspect.

[149] Weatherly L.M., Gosse J.A. (2017). Triclosan exposure, transformation, and human health effects. J. Toxicol. Environ. Health Part B Crit. Rev..

[150] Ma R., Tang N., Feng L., Wang X., Zhang J., Ren X., Du Y., Ouyang F. (2021). Effects of triclosan exposure on placental extravillous trophoblast motility, relevant IGF2/H19 signaling and DNA methylation-related enzymes of HTR-8/SVneo cell line. Ecotoxicol. Environ. Saf..

[151] Parolini M., De Felice B., Mondellini S., Caprioli M., Possenti C.D., Rubolini D. (2021). Prenatal exposure to triclosan induced brain telomere shortening in a wild bird species. Environ. Toxicol. Pharmacol..

[152] Rosen E.M., Muñoz M.I., McElrath T., Cantonwine D.E., Ferguson K.K. (2018). Environmental contaminants and preeclampsia: A systematic literature review. J. Toxicol. Environ. Health.

[153] Canudas S., Becerra-Tomás N., Hernández-Alonso P., Galié S., Leung C., Crous-Bou M., De Vivo I., Gao Y., Gu I., Meinilä C. (2020). Mediterranean Diet and Telomere Length: A Systematic Review and Meta-Analysis. Adv. Nutr..

[154] Zhang R., Du J., Xiao Z., Jiang Y., Jin L., Weng Q. (2022). Association between the peripartum maternal and fetal telomere lengths and mitochondrial DNA copy numbers and preeclampsia: A prospective case–control study. BMC Pregnancy Childbirth.

